# CaCO_3_ powder-mediated biomineralization of antigen nanosponges synergize with PD-1 blockade to potentiate anti-tumor immunity

**DOI:** 10.1186/s12951-023-01870-x

**Published:** 2023-04-07

**Authors:** Runping Su, Jingjing Gu, Juanjuan Sun, Jie Zang, Yuge Zhao, Tingting Zhang, Yingna Chen, Gaowei Chong, Weimin Yin, Xiao Zheng, Bingbing Liu, Li Huang, Shuangrong Ruan, Haiqing Dong, Yan Li, Yongyong Li

**Affiliations:** 1 Shanghai Skin Disease Hospital, The Institute for Biomedical Engineering & Nano Science, School of Medicine, Tongji University, Shanghai, 200092 P. R. China; 2 Key Laboratory of Medical Molecular Virology (MOE/NHC/CAMS), School of Basic Medical Sciences, Fudan University, Shanghai, 200032 China; 3 Institute of Acoustics, School of Physics Science and Engineering, Tongji University, Shanghai, 200092 China

**Keywords:** Nanovaccine, CaCO_3_ powder, Biomineralization, Antigen-presenting cells, Antigen-specific CD8^+^ T cell responses

## Abstract

Antigen self-assembly nanovaccines advance the minimalist design of therapeutic cancer vaccines, but the issue of inefficient cross-presentation has not yet been fully addressed. Herein, we report a unique approach by combining the concepts of “antigen multi-copy display” and “calcium carbonate (CaCO_3_) biomineralization” to increase cross-presentation. Based on this strategy, we successfully construct sub-100 nm biomineralized antigen nanosponges (BANSs) with high CaCO_3_ loading (38.13 wt%) and antigen density (61.87%). BANSs can be effectively uptaken by immature antigen-presenting cells (APCs) in the lymph node upon subcutaneous injection. Achieving efficient spatiotemporal coordination of antigen cross-presentation and immune effects, BANSs induce the production of CD4^+^ T helper cells and cytotoxic T lymphocytes, resulting in effective tumor growth inhibition. BANSs combined with anti-PD-1 antibodies synergistically enhance anti-tumor immunity and reverse the tumor immunosuppressive microenvironment. Overall, this CaCO_3_ powder-mediated biomineralization of antigen nanosponges offer a robust and safe strategy for cancer immunotherapy.

**Supplementary Information:**

The online version contains supplementary material available at 10.1186/s12951-023-01870-x.

## Introduction

Nanotechnology has been extensively applied to overcome the limited immunogenicity of vaccines [1]. In contrast to those antigen conjugations onto or encapsulated into nanoparticles, nanovaccine with antigen self-assembly is conducive to improving immunogenicity by taking advantage of multi-copy display effects like viruses [2–4]. Our previous study demonstrated that the minimalist nanovaccine constituted from near whole antigen (up to 97 wt%) showed benefits in improving immunogenicity, especially for a humoral immune response [5]. However, the tumor therapeutic effect is limited by the low efficiency of cross-presentation. Cross-presentation is the key to the activation of cytotoxic T lymphocytes (CTLs), which is the bottleneck in developing therapeutic vaccines [1]. The solution to these challenges is integrating components that enhance cross-presentation into the vaccine nanostructure without compromising antigen density.

Adjuvants, in the context of vaccines, are defined as components capable of enhancing cross-presentation and shaping antigen-specific immune responses [6]. Notably, calcium adjuvants have gained tremendous attention in promoting cross-presentation [7–10]. Generally, calcium adjuvants are introduced into vaccine design through a one-pot biomineralization strategy (calcium source in solution). However, the integration of calcium adjuvants like Calcium carbonate (CaCO_3_) is easy to lead to large particle sizes (~ 500 nm) [7], which are not favorable for lymph node transportation of vaccine and antigen cross-presentation. The underlying mechanism of the one-pot biomineralization method is probably associated with the fast kinetic reaction of Ca^2+^ and CO_3_^2−^, leading to random and quick biomineralization [11–13]. In addition, the uncontrolled quick biomineralization tends to embed the antigen inside and thus adversely affects the exposure of antigen display. Therefore, it is critical to realize controlled biomineralization by reducing the reaction kinetics and slowing the mineralization process. Inspired by the control of low radical concentration during atom transfer radical polymerization (ATRP)[14, 15], a dynamic continuous process is proposed to precisely control CaCO_3_ biomineralization, mediated by CaCO_3_ powder in a liquid-solid reaction system.

**Scheme 1:**
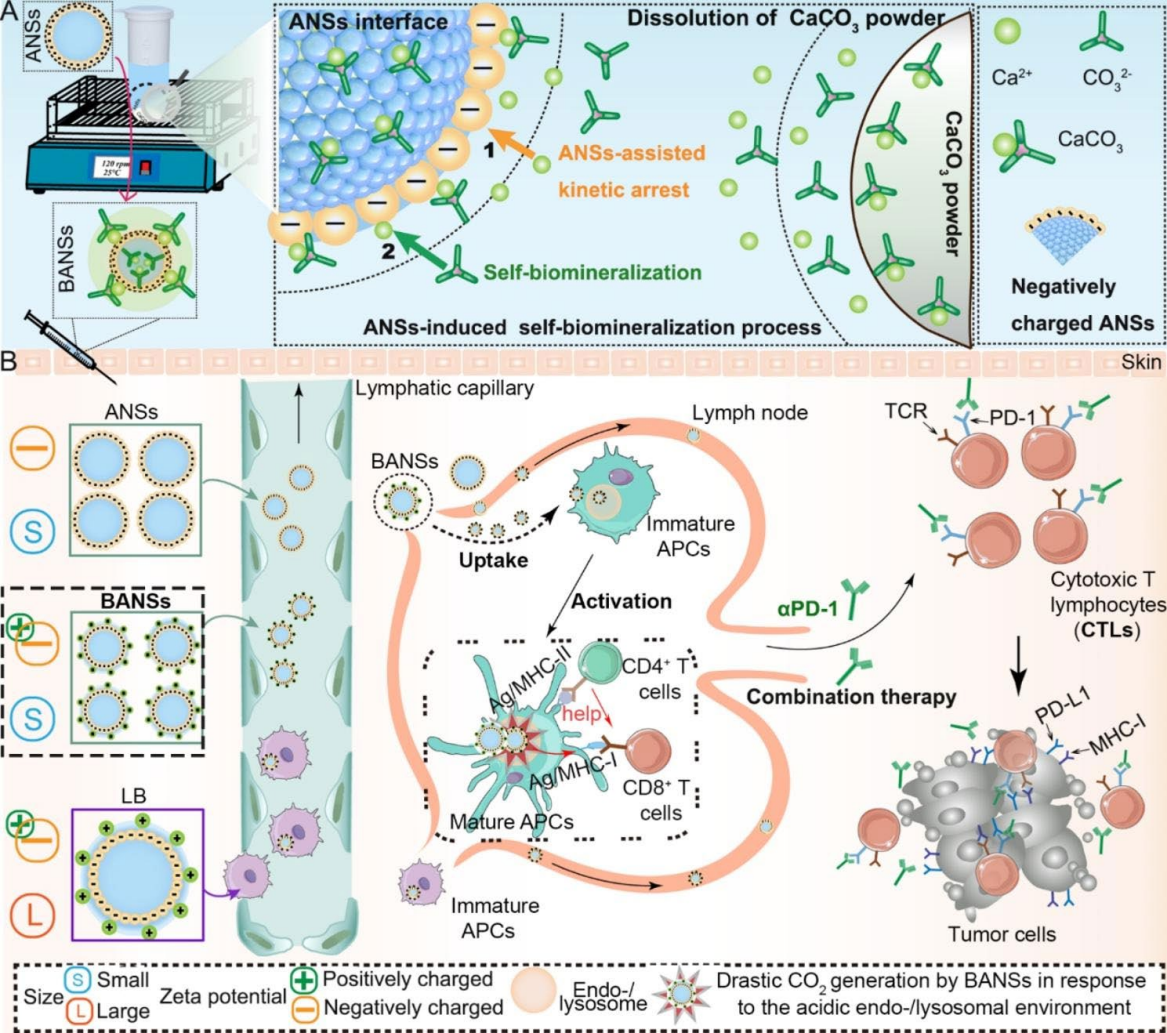
Schematic illustration of antigen nanosponges (ANSs) as templates for biomineralization and cancer immunotherapy mechanisms. (**A**) Schematic representation of a dynamically continuous biomineralization strategy. Based on this strategy, we successfully constructed sub-100 nm biomineralized antigen nanosponges (BANSs) and large sizes of BANSs (LB). (**B**) After subcutaneous injection of small-sized and less negatively charged BANSs, it could be efficiently uptaken by immature APCs and generated drastic carbon dioxide (CO_2_) in response to the acidic endo-/lysosomal environment for antigen cross-presentation to CD8^+^ T cells through the major histocompatibility complex class I (MHC I) pathway. The interaction of the T cell receptor (TCR) and the peptide-MHC-I complex on the tumor cell surface regulates CTLs response and tumor-killing ability. A combination of BANSs with anti-PD-1 antibody (αPD-1) generated synergistic effects that potentiated antitumor immunity

In this study, the vaccine nanostructure was called antigen nanosponges and the strategy of CaCO_3_ powder-mediated biomineralization in antigen nanosponges was developed to precisely control the process of CaCO_3_ biomineralization. The CaCO_3_ loading was elaborately tuned by adjusting the reaction time in a low and constant calcium ion concentration (0.65 mM). Based on this strategy, we successfully constructed sub-100 nm biomineralized antigen nanosponges (BANSs) with high CaCO_3_ loading (38.13 wt%) and antigen density (61.87%). BANSs were effectively uptaken by immature antigen-presenting cells (APCs) in the lymph nodes (LNs) upon subcutaneous injection. Achieving efficient spatiotemporal coordination of antigen cross-presentation and immune effects, BANSs induced the production of CD4^+^ T helper (Th1) cells and CTLs, resulting in effective tumor growth inhibition. BANSs combined with anti-PD-1 antibody (αPD-1) synergistically enhanced anti-tumor immunity and reverse the tumor immunosuppressive microenvironment (Scheme 1).

## Results and discussion

### CaCO_3_ powder-mediated controlled biomineralization of ANSs

We first investigated the feasibility of ANSs could be acted as templates for continuous biomineralization. Based on the previously established method [9], the constructed ANSs were smaller than 40 nm and negatively charged (Figure S1A, B, Supporting Information), and their carboxyl groups (C = O stretching) (Figure S1C, Supporting Information) provided chelating sites for Ca^2+^, which determined that ANSs could be acted as biomineralized templates. In addition, ANSs formed by disulfide bonds, hydrogen bonds, and intermolecular hydrophobic interactions further improved the stability of ANSs against alien intervention [5]. Different particle sizes of ANSs were prepared by adjusting the reaction time (Figure S1D, Supporting Information), and the particle sizes of ANSs within 100 nm were achieved (Figure S1E, F, Supporting Information).

Protein-based biomineralization processes could be affected by regulating the Ca^2+^ concentration, pH, and surface properties of protein [16, 17]. Based on the fact that pH can affect both the Ca^2+^ release from CaCO_3_ powder and the surface properties of the protein, we hypothesized that pH was the key parameter that regulates this process in our study. Instead of a water-soluble calcium salt solution, we used a water-insoluble solid calcium source (calcium carbonate powder) in the mineralization process. The purpose is to maintain a constant and trace concentration of calcium in the solution, in order to control the smooth growth of nanoparticles to a reasonable size. Therefore, the pH range of 4.5-7 was employed to regulate the concentration of calcium to ensure proper growth kinetics and thus results in the desired size of the nanoparticle [18].

**Fig. 1:**
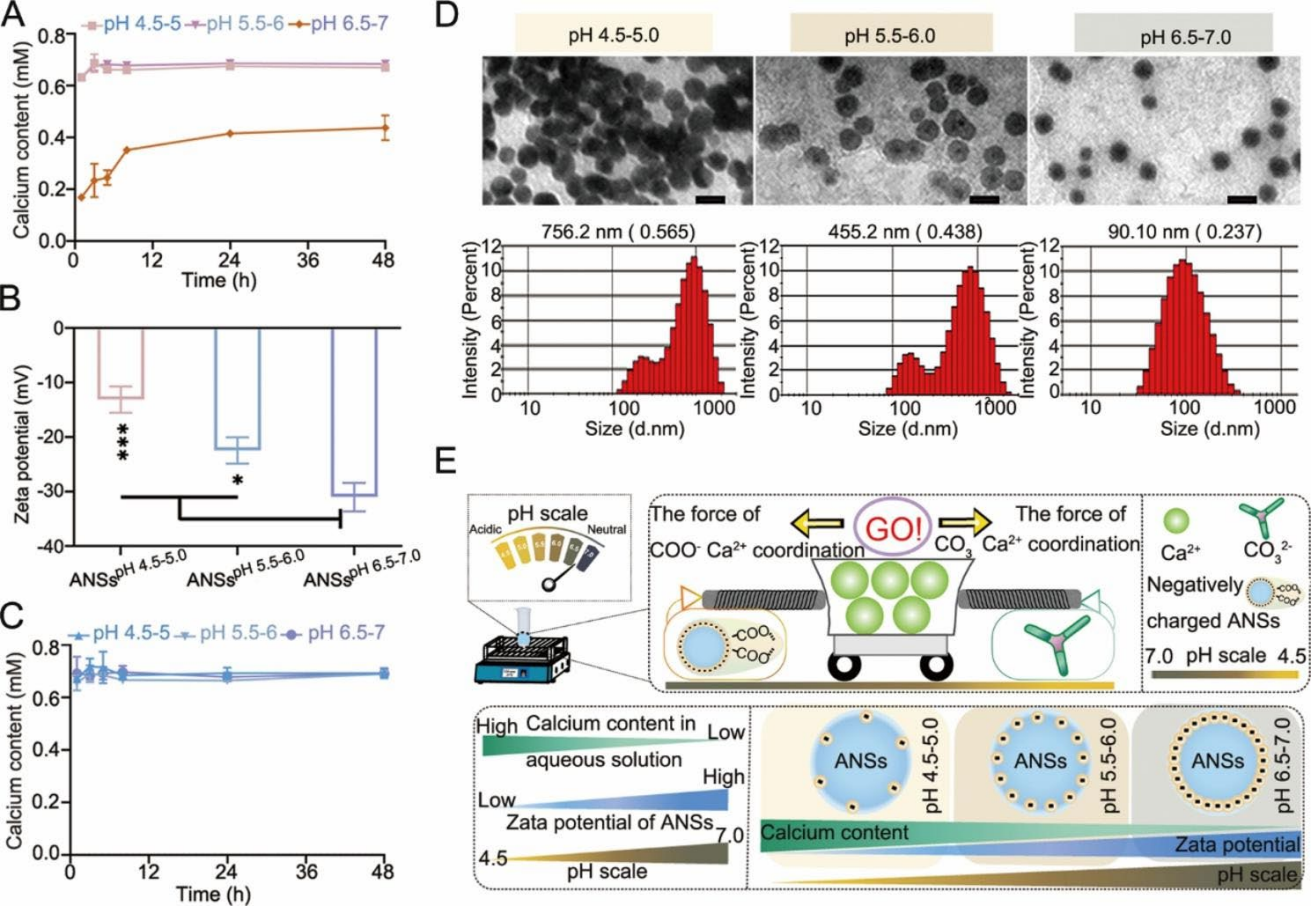
CaCO_3_ powder-mediated controlled biomineralization of ANSs. (**A**) The Ca^2+^ generation from CaCO_3_ powder in various pH aqueous solutions. (**B**) Surface zeta potential of ANSs at different pH ranges. (**C**) The Ca^2+^ generation from CaCO_3_ powder in various pH of ANSs solutions. (**D**) Representative transmission electron microscopy (TEM) and particle size distribution of BANSs at different pH ranges. Scale bars: 0.1 μm. (**E**) Schematic drawings comparing surface zeta potential of ANSs and Ca^2+^ generation from CaCO_3_ powder in various pH aqueous solutions

As shown in Fig. 1A and B, the Ca^2+^ concentration in the aqueous solution was the lowest, and the surface zeta potential of ANSs was the highest at pH 6.5-7.0 compared to other pH ranges. As expected, the Ca^2+^ concentration (0.65 mM) in the ANSs solution was highly consistent at different pH ranges and at various times (Fig. 1C). As shown in Fig. 1D, the particle size and dispersity of BANSs varied significantly at different pH values. Specifically, at pH 4.5-5.0 and pH 5.5-6.0, the particle size of BANSs was 756.2 nm (with a dispersity of 0.565) and 455.2 nm (with a dispersity of 0.438), respectively, indicating that BANSs tended to aggregate at pH 4.5-6.0. However, at pH 6.5-7.0, BANSs showed a much smaller particle size of 90.10 nm (with a dispersity of 0.237), indicating that they were uniformly dispersed and much less prone to aggregation. In addition, too high or too low temperatures and shaking speeds were not conducive to achieving controlled and stable biomineralization of ANSs (Figure S2 Supporting Information). The above results indicated that continuous biomineralization was achieved by setting the shaking conditions at 25 °C with the speed of 120 rpm in the ANSs solution of pH 6.5-7.0.

Further analysis of the preparation process of BANSs showed that both carboxyl groups (COO^−^) and carbonate groups (CO_3_^2−^) coordinated with Ca^2+^ in this process, and the adequate adjustment of pH may adjust the different coordination (Fig. 1E). Two effects were achieved by adjusting the pH range: maintaining a constant Ca^2+^ concentration in the ANSs’ solution; the other is determining that ANSs had a higher negative charge. ANSs captured Ca^2+^ through the carboxyl groups and formed CaCO_3_ with CO_3_^2−^ from the solution. Once the Ca^2+^ concentration was lower than the saturated Ca^2+^ concentration, the CaCO_3_ powder would release more Ca^2+^ to maintain the saturated concentration and promoted the growth of CaCO_3_ crystals. Thus, the process of CaCO_3_ biomineralization was maintained continuously and highly controllable.

### Design and characterization of BANSs

**Fig. 2:**
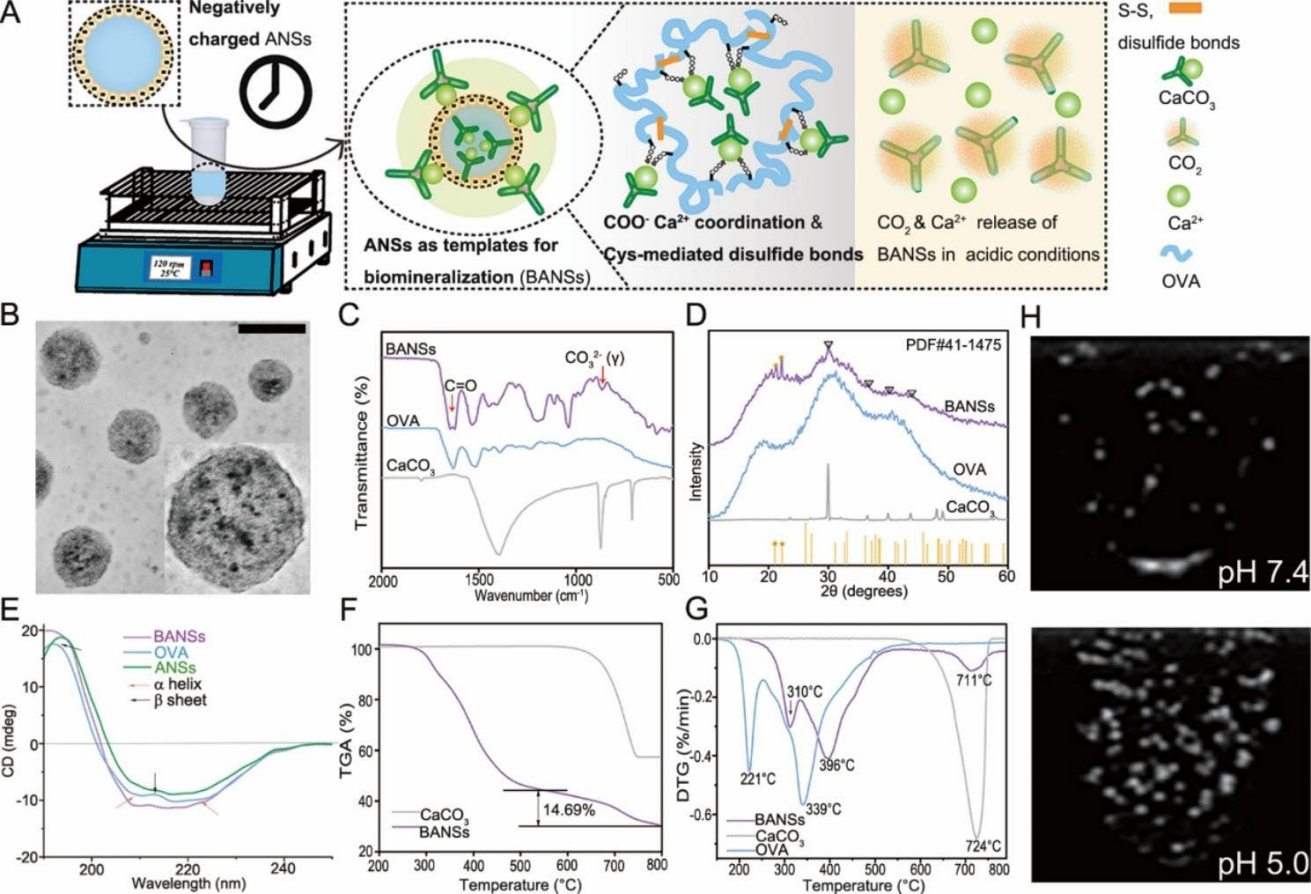
Design and characterization of BANSs. (**A**) Schematic illustration of BANSs preparation and their decomposition in an acidic microenvironment. (**B**) TEM images of BANSs (scale bar, 0.1 μm). (**C**) Fourier-transform infrared spectroscopy (FT-IR) spectra of BANSs, OVA, and CaCO_3_ (**D**) X-ray powder diffraction (XRD) pattern of the BANSs, OVA, and pristine CaCO_3_. (**E**) Circular dichroism (CD) spectrum of OVA, ANSs, and BANSs. (**F**) Thermal gravimetric analysis (TGA) of CaCO_3_ and BANSs. (**G**) Derivate TGA DTG curves of BANSs, OVA, and CaCO_3_. (**H**) The CO_2_ generated by BANSs in neutral and acidic environments was measured using an 8 MHz linear transducer

Based on this strategy, we successfully constructed BANSs (Fig. 2A). The BANSs had a distinct mineralized layer with good dispersion (Fig. 2B). The average particle size of BANSs was around 88 nm, and the surface zeta potential of BANSs was − 16 mV (Figure S3, Supporting Information). The energy-dispersive x-ray spectroscopy (EDX) analysis (Figure S4, Supporting Information) showed the presence of C, Ca, O, and S elements in BANSs. In addition to the characteristic peak of OVA at 1644 cm^− 1^ (C = O), the BANSs showed a characteristic peak with the carbonate group at 875 cm^− 1^ (Fig. 2C). As shown in the x-ray powder diffraction (XRD) of Fig. 2D, the diffraction peak of the BANSs coincided perfectly with the diffraction peak of the CaCO_3_ standard powder diffraction file card (#41-1475) (aragonite) and the CaCO_3_ powder (calcite), indicating the presence of calcite and aragonite crystalline forms. As shown in Fig. 2E, the intensity of the characteristic peaks of the protein α-helix and random coil structures was significantly increased in BANSs compared to OVA and ANS. This suggested that biomineralization increases the stability of the α-helix and random coil structures, possibly due to intermolecular interactions and interactions between the -COOH groups and Ca^2+^[19]. Thermal gravimetric analysis (TGA) showed a 14.69% mass loss of the BANSs (Fig. 2F). Figure 2G showed the DTG peak of BANSs (711 °C) was close to the peak position of the DTG peak of CaCO_3_ (724 °C), which indicated that CaCO_3_ is effectively mineralized in the ANSs [20]. Inductively Coupled Plasma Optical Emission Spectrometer (ICP-OES) determined the CaCO_3_ content to be 38.13 wt%.

To determine whether the decomposition of BANSs was related to the acidic environment of endo-/lysosomes, we next investigate whether BANSs released Ca^2+^ and CO_2_ in an acidic environment. The release of Ca^2+^ at pH 5.0 was significantly higher than that at pH 7.4 (Figure S5, Supporting Information). The results showed that BANSs rapidly produced CO_2_ bubbles in a simulated endo-/lysosomal environment (pH 5.0) (Fig. 2H).

### Comparison of the Conventional one-pot and continuous biomineralization processes

**Fig. 3:**
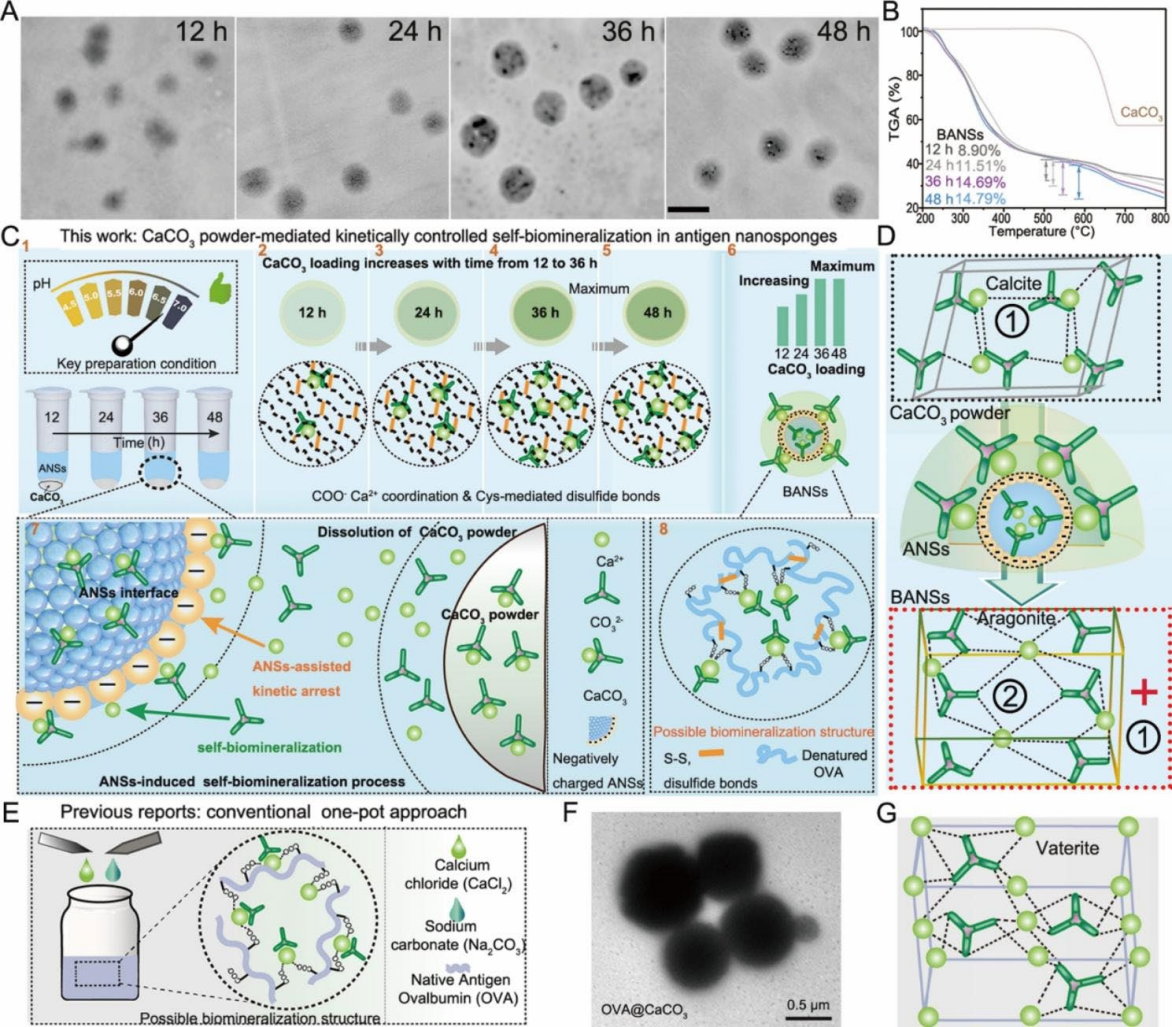
Comparison of the conventional one-pot and continuous biomineralization approach. (**A**) TEM of BANSs in ANSs solutions (pH 6.5-7) at various times. Scale bars: 0.1 μm. (**B**) TGA of CaCO_3_ and BANSs for various times. (**C**) Schematic representation of continuous biomineralization approach and the possible molecular structure of BANSs. (**D**) Schematic of the crystal structures of CaCO_3_ powder and BANSs based on the XRD pattern in Fig. 2D. (**E**) Schematic illustration of conventional one-pot approach. (**F**) TEM images of OVA@CaCO_3_ (scale bar, 0.5 μm). (**G**) Schematic illustration of the crystallographic structure of OVA@CaCO_3_.

The continuous (solid-state calcium source) biomineralization processes were then detected by TEM, dynamic light scattering (DLS), and TGA at pH 4.5-7.0 at different time points. As shown in Fig. 3A, the prepared BANSs were well dispersed and stable over time. In addition, the particle size of BANSs was smaller than 100 nm during 48 h (Figure S6A, Supporting Information). The zeta potential of the ANSs was -32.3 mV, while that of the BANSs shifted to -16 mV after 36 h and showed no obvious changes within next 12 h (Figure S6B, Supporting Information). Further, the mass loss of BANSs increased as time increased and BANSs achieved the maximum loading of CaCO_3_ at 36 h (Fig. 3B).

In the continuous biomineralization approach, the water-soluble calcium salt solution in the conventional one-pot method was replaced by a water-insoluble solid calcium source (CaCO_3_ powder) **(**Fig. 3C, E). The current strategy possesses several fundamental differences when compared to the conventional (solution-based) CaCO_3_ biomineralization vaccine, such as: (1) solid calcium source CaCO_3_ powder instead of calcium salt solution; (2) the absence of counter ions from soluble calcium salts, such as Cl^−^ from CaCl_2_; (3) the continuous nature of the biomineralization process versus discrete nature in conventional method; (4) time-dependent control over CaCO_3_ loading, as shown in Fig. 3B; (5) smaller particle size of the CaCO_3_ biomineralized vaccines, i.e., 500 nm OVA@CaCO_3_ (Fig. 3F) vs. less than 100 nm BANSs (Fig. 3A); (6) the transformation of the CaCO_3_ crystal structures (Fig. 3D,G), BANSs consisted of two CaCO_3_ crystalline forms (calcite and aragonite) (Figs. 2D and 3D), whereas the crystalline form of CaCO_3_ in OVA@CaCO_3_ was vaterite phase as reported [7]; The CaCO_3_ crystal structure was related to its stability, the CaCO_3_ crystalline forms of calcite (triangles structure) and aragonite (rhombohedral crystal system) were stable under ambient conditions [13, 21].

### BANSs promote cytosolic delivery of antigens, enhance the maturation and cross-presentation

**Fig. 4:**
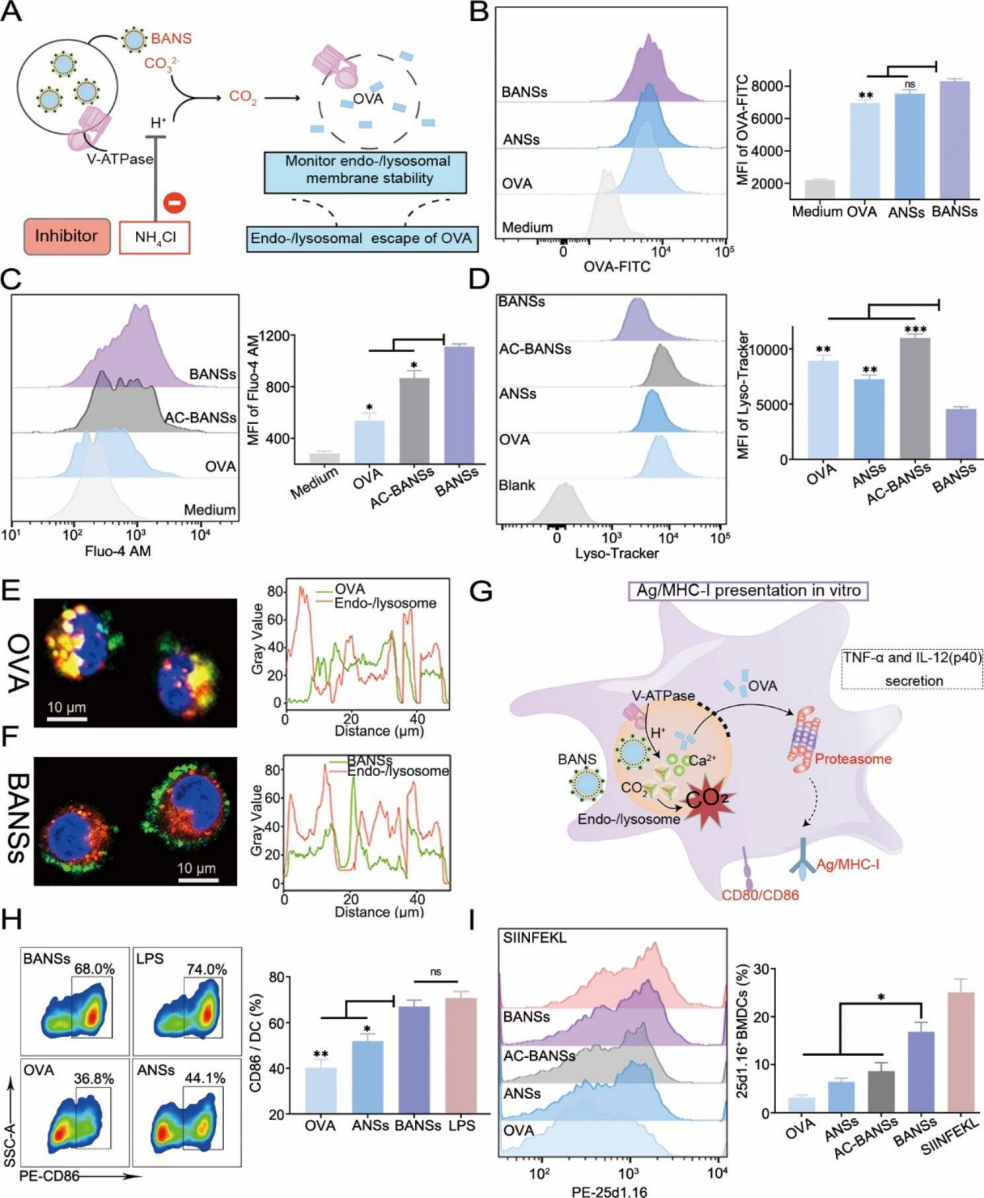
BANSs promote the cytosolic delivery of antigens and enhance maturation and cross-presentation. (**A**) Schematic diagram of cytosolic delivery of antigens. (**B**) Phagocytosis efficiency of DC2.4 on different vaccine formulations by flow cytometry. (**C**) Flow cytometry analysis of intracellular Ca^2+^ levels. (**D**) Flow cytometry analysis of endo-/lysosomal membrane instability. CLSM analysis of OVA (**E**) and BANSs (**F**) about endosome/lysosome co-localization Endo-/lysosomes (red) were stained with Lyso-Tracker Red. Nuclei (blue) were stained with Hoechst 33,342. OVA and BANSs were labeled with FITC (green). Scale bars are 10 μm. (**G**) Schematic illustration of BMDC maturation and antigen presentation by BANSs. (**H**) BANSs promote the maturation of BMDCs. (**I**) BANSs promote the in vitro cross-presentation of BMDC. Data are shown as the mean ± SD (*n* = 3). (ns, not significant, *P < 0.05, **P < 0.01 and ***P < 0.001)

According to the schematic illustration (Fig. 4A), we first demonstrated whether BANSs could promote the cytosolic delivery of antigens, a critical step for cross-presentation. Based on the cytotoxicity, dosage of BANSs below 125 µg/mL was selected for subsequent cell experiments (Figure S7, Supporting Information). At 6 h, no obvious difference in the cell internalization efficiency of OVA in the BANSs group was compared to the ANSs group (Fig. 4B). However, the uptake level of antigen by cells in the BANSs group increased in a concentration-dependent manner, reaching the maximum internalization efficiency at 100 µg/mL (Figure S8, Supporting Information).

At the cellular level, CO_2_ production was indirectly demonstrated by examining the release of Ca^2+^. To determine whether the release of Ca^2+^ is related to the acidic environment of endo-/lysosomes, we employed the acidification inhibitor NH_4_Cl to alkalize the acidic environment of endo-/lysosomes [22]. It was found that the intracellular Ca^2+^ level in DC2.4 cells in the for BANSs group was higher compared with those under OVA and NH_4_Cl treatment (Fig. 4C), indicating that BANSs controllably released Ca^2+^ and CO_2_ in an acidic endo-/lysosomal environment. Flow cytometry detected endo-/lysosomal stability by quantifying the relative amount of Lyso-Tracker Red in endo-/lysosomes [23]. The fluorescence intensity produced by the BANSs group was significantly lower than that of OVA and ANSs, whereas increased significantly after NH_4_Cl treatment (Fig. 4D). In addition, the CLSM images clearly showed the co-localization antigen with endo-/lysosomes. Compared to the OVA with a PPC value of 0.86 (Fig. 4E), the co-localization of the Lyso-Tracker signal with the FITC signal was not apparent in the cells of the BANSs group with a PPC value of 0.46, indicating that BANSs were able to escape endo-/lysosomes (Fig. 4F).

Usually, the efficiency of antigen cross-presentation was influenced by the maturation of APCs (Fig. 4G)[24]. Compared to OVA and ANSs, BANSs promoted the expression of the co-stimulatory factor CD86 (Fig. 4H) and triggered increased secretion of TNF-α and IL-12p40 (Figure S9, Supporting Information). Lipopolysaccharide (LPS), a ligand of Toll-Like Receptor 4, is a potent maturation stimulator that can be employed as a positive control [25]. The LPS group resulted in a non-significant difference in the expression of CD86 with BANSs groups. The cross-presentation efficiency was quantitatively evaluated by examining H-2Kb-SIINFEKL complexes on BMDC surfaces labeled with the monoclonal antibody 25d1.16. It was found that BANSs contributed to a 5.3-fold, 2.6-fold, and 1.9-fold increase in 25d1.16 positive cells compared to OVAs, ANSs, and AC-BANSs, respectively (Fig. 4I). The results confirmed that CaCO_3_ biomineralization in ANSs enhanced the maturation and antigen cross-presentation in vitro.

### Spatiotemporal lymph nodes distribution of nanovaccines and subsequent immune responses

**Fig. 5:**
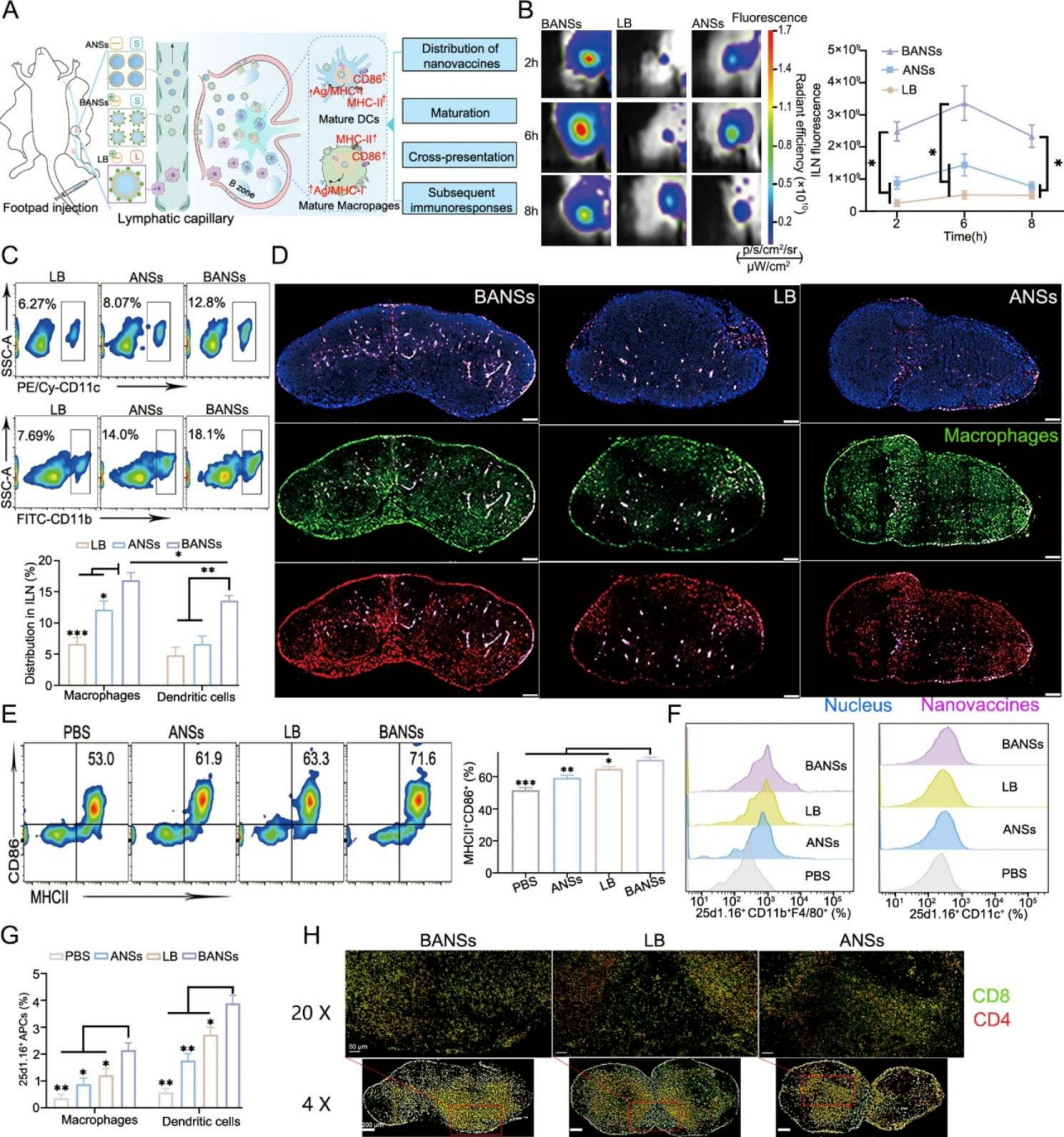
Characterizations of lymph node distribution and subsequent immune responses. (**A**) Scheme of vaccines with different sizes or surface charges accumulation in inguinal lymph nodes (ILNs) and subsequent immune responses in vivo. (**B**) In vivo imaging and fluorescence statistics. (**C**) Flow cytometry detection of vaccine uptake by dendritic cells (DCs) and macrophages (Mø) in ILNs. (**D**) Fluorescence images of ILNs frozen sections. Scale bars are 200 μm. (**E**) Flow cytometric detection of the expression levels of surface molecules MHC-II and CD86 in DCs. (**F**) Representative flow cytometry histograms of Kb-SIINFEKL expression in DCs and macrophages. (**G**) Detection of in vivo antigen cross-presentation levels of DCs and Mø by flow cytometry. (**H**) Immunofluorescence analysis of lymph node sections at day 6 postvaccination. Data are shown as the mean ± SD (*n* = 3) from three independent animals. (*P < 0.05, **P < 0.01 and ***P < 0.001)

The findings suggested that the physicochemical properties (e.g., surface charges and size) of vaccine delivery systems could affect their LNs distribution and subsequent immune responses [26, 27]. Thus, three vaccine delivery systems were established to explore their distribution in lymph nodes (Fig. 5A), namely BANSs (-16 mV, 88 nm), ANSs (-31 mV, 84 nm), and LB (-20 mv, 281 nm) (Figure S10, Supporting Information), which were labeled with BODIPY to facilitate the optical imaging observation in vitro (Figure S11, Supporting Information). According to Fig. 5B, BANSs quickly reached popliteal lymph nodes (PLNs) within 6 h, while LB showed only weak fluorescence signals. In addition, the quantification of the fluorescence signal intensity showed that the antigen accumulation in the BANSs group was 6.6-fold and 2.3-fold higher than that in the LB and ANSs groups at 6 h, respectively. We found that the smaller size and less negative surface charge of BANSs may have made them more easily taken up and transported by lymphatic vessels than larger or more negatively charged particles such as ANSs and LB. Specifically, larger-size carriers (> 200 nm) may be trapped in the extracellular matrix and suffer from lytic malabsorption. Smaller nanoparticles (< 100 nm) are generally more easily taken up by lymphatic vessels and transported to the lymph node. In addition, more negatively charged nanoparticles may be repelled by the negatively charged lymphatic endothelial cells and are less likely to be taken up [28, 29].

Moreover, dendritic cells (DCs) and macrophages (Mø) had a higher uptake capacity for BANSs than LBs and ANSs. Notably, CD11b^+^Mø was significantly more capable of uptake of BANSs than CD11c^+^DCs (Fig. 5C). Furthermore, although both BANSs and LB were found in the central paracortex of LNs, the enrichment of BANSs was much higher than that of LBs. In contrast, ANSs were more enriched in the subcapsular sinus than in the central paracortex of LNs (Fig. 5D).

Further study demonstrated that BANSs significantly increased the expression levels of MHC-II and CD86 on the surface of DCs and Mø compared with that in PBS and ANSs groups (Fig. 5E and Figure S12, Supporting Information). In addition, the number of 25d1.16-positive DCs and Mø in the BANSs group was significantly higher than that in the PBS, ANSs, and LB groups (Fig. 5F, G). To investigate the effect of different vaccine delivery systems on the immune response in lymph nodes, the inguinal lymph nodes (ILNs) were isolated for immunofluorescence staining of CD4 T cells and CD8 T cells. Compared with LB and ANSs groups, the number of CD8 T and CD4 T cells was increased in the BANSs group. In addition, we found a significant migration of CD8 T and CD4 T cells toward the germinal center in the BANSs group (Fig. 5H). In addition, Fig. 5H showed a distribution of CD4 and CD8 co-localization in the BANs group compared to the control LBs and ANSs.

### The ability of BANSs to inhibit tumor growth

**Fig. 6:**
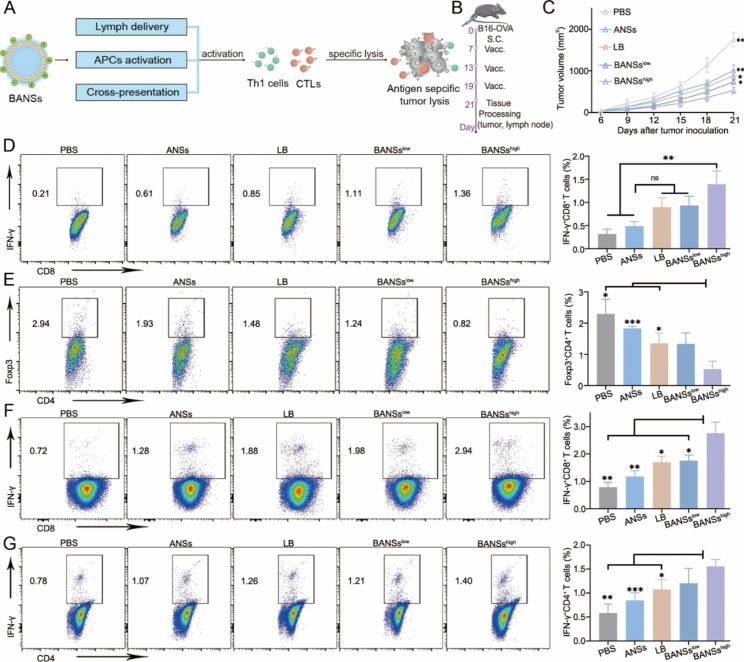
The ability of BANSs to inhibit tumor growth. (**A**) Schematic illustration of the therapeutic mechanism of BANSs. (**B**) Schematic illustration of BANSs study protocol. (**C**) Tumor growth curve. (**D**) Detection of intratumoral IFN-γ^+^CD8^+^ T cells by flow cytometry. (**E**) Detection of intratumoral Foxp3^+^CD4^+^ T cells by flow cytometry. (**F**) Detection of IFN-γ^+^CD8^+^ T cells in dLNs by flow cytometry. (**G**) Detection of IFN-γ^+^CD4^+^ T cells in dLNs by flow cytometry. Data are shown as the mean ± SD from four or five independent animals. (ns, not significant, *P < 0.05, **P < 0.01 and ***P < 0.001)

BANSs improved the cross-presentation of exogenous antigens and subsequent immune response, which was essential for tumor suppression, prompting us to evaluate the antitumor effects of BANSs (Fig. 6A). C57BL/6 mice were vaccinated with PBS, ANSs, LB, BANSs^low^ (Nanovaccines loaded with low contents of calcium carbonate), and BANSs^high^ (Nanovaccines loaded with high contents of calcium carbonate) on days 7, 9, and 13 after tumor cell inoculation (Fig. 6B). BANSs^high^ showed a better effect in inhibiting B16-OVA tumor growth than the other vaccine groups (Fig. 6C).

To investigate the mechanism of tumor growth inhibition, we analyzed T cells in the tumor and draining lymph nodes (dLNs) by flow cytometry. Analysis of INF-γ^+^CD8^+^ T cells in the tumor showed that BANSs^high^ generated a more distinct IFN-γ^+^CD8^+^ T cells response than PBS and ANSs (Fig. 6D). The proportion of Foxp3^+^CD4^+^ regulatory T cells (Tregs) in tumor tissues of mice treated with BANSs^high^ was significantly lower than that of the mice treated with PBS, ANSs, and LB (Fig. 6E). In addition, the BANSs^high^ produced more IFN-γ^+^CD8^+^ T cells and IFN-γ^+^CD4^+^ T cells than PBS, ANSs, and LB in the dLNs (Fig. 6F, G). These results indicated that BANSs^high^ had advantages in the therapeutic effect, and thus the concentration of BANSs^high^ was used for the follow-up study.

### Combination of BANS and αPD-1 for cancer immunotherapy

**Fig. 7:**
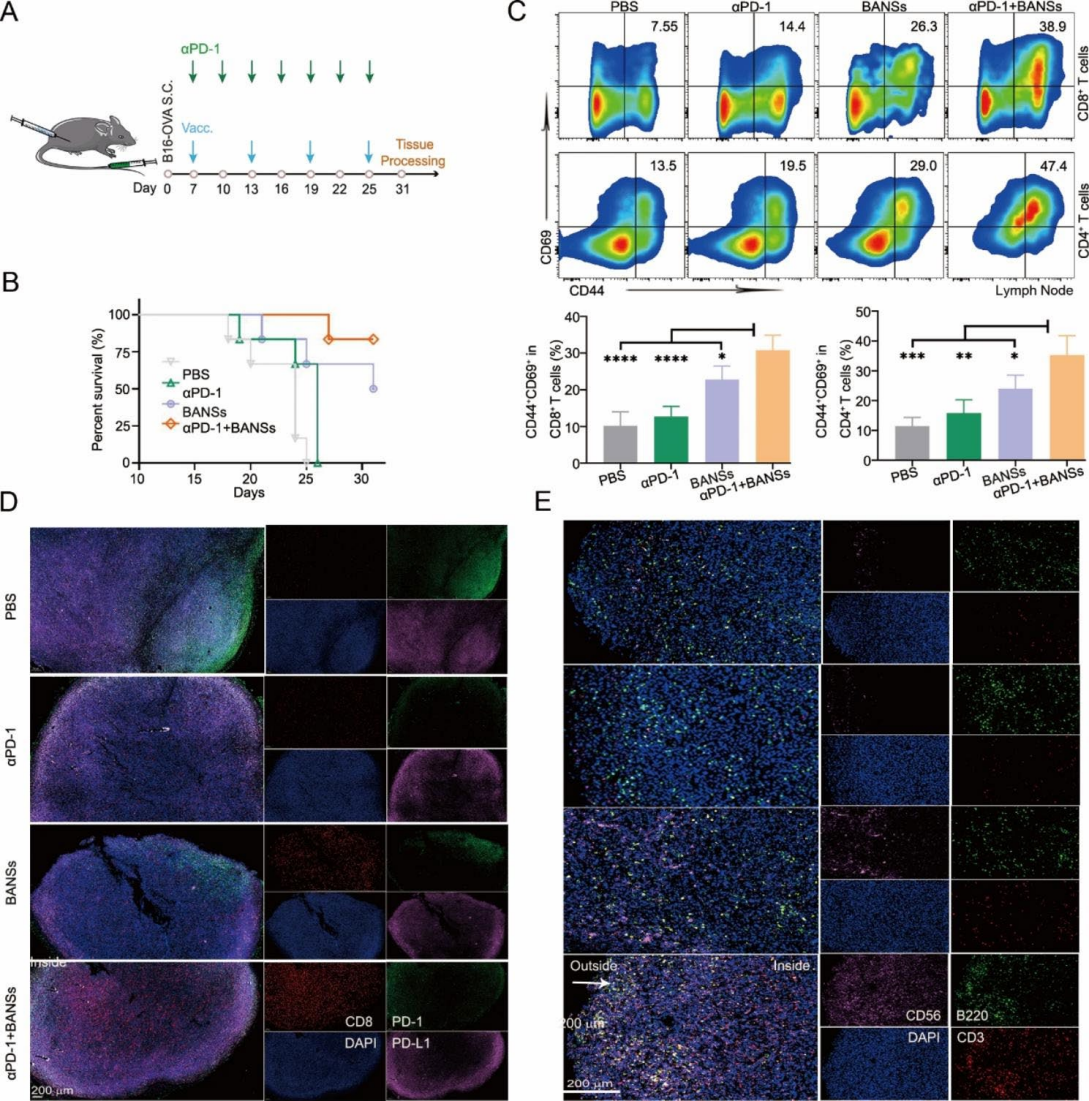
Combination of BANS and αPD-1 for cancer immunotherapy. (**A**) Schematic illustration of BANSs in combination with the αPD-1 study protocol. (**B**) Survival curve of C57BL/6 mice. (**C**) Detection of activated T cells in LNs by flow cytometry (*n* = 4 to 6 mice per group). (**D**) Multiplex immunohistochemistry (mIHC) analysis of tumor immunosuppressive environment. (**E**) mIHC analysis of tumor immune environment. (ns, not significant, *P < 0.05, **P < 0.01, ***P < 0.001 and ****P < 0.0001)

BANSs were combined with αPD-1 further to assess the therapeutic effect (Fig. 7A). BANSs combined with αPD-1 effectively inhibited tumor growth (Figure S13, Supporting Information). We set animal death according to the natural mortality and humanitarian endpoints in mice (subcutaneous tumor volume to 2000 mm^3^) [30]. Through analysis of survival curves, we found that αPD-1 combined with BANSs improved the survival rate of mice compared with other groups (Fig. 7B). Activation of T cells in lymph nodes and spleen by αPD-1 + BANSs was analyzed by flow cytometry. Studies have shown that activated T cells expressed more cell surface proteins, such as CD44 and CD69, which distinguished them from the initial T cells [31]. BANSs combined with αPD-1 were able to activate more CD44^hi^CD69^hi^CD8^+^ T cells and CD44^hi^CD69^hi^CD4^+^ T cells compared to other agents (Fig. 7C, Figure S14, Supporting Information). Recent studies have shown that the density of tumor-infiltrating T cells, NK cells, and B lymphocytes is critical for cancer immunotherapy and patient survival [32, 33]. Subsequently, the tumor microenvironment was assessed by mIHC. As shown in Fig. 7D, significantly lower levels of PD-1 were observed in the αPD-1 + BANSs group compared with the other groups, and the number of CD8^+^ T cells was significantly increased in the αPD-1 + BANSs group. In addition, the number of B cells, natural killer cells (NK), and T cells at the tumor site were significantly increased in the αPD-1 + BANSs group compared with the other groups (Fig. 7E). The above results suggested that combining BANSs with αPD-1 effectively activated T cells and provided immediate anti-tumor immunity.

During the vaccination period, we found no significant weight loss in mice (Figure S15A, Supporting Information). In addition, there were no differences in serum biochemistry and the HE staining of major organs between treated and normal control mice (Figure S15B-F, Figure S16, Supporting Information), with clearly visible nuclei and well-defined stained nucleoplasm, suggesting the biological safety of these treatments.

## Conclusion

The constructed BANSs achieved spatiotemporal coordination of antigen cross-presentation and immune response within different APCs of lymph nodes. In addition, BANSs combined with αPD-1 synergistically enhanced anti-tumor immunity and reversed the tumor immunosuppressive microenvironment. In conclusion, we presented a dynamically continuous biomineralization strategy to prepare nanoscale mineralized vaccines with high CaCO_3_ loading. A relevant feature of the current method is its ability to control the CaCO_3_ loading by adjusting the reaction time. The dynamic process presented herein provides new insights into the nature of mineral ion-protein biomineralization that may find broad application in biomedicine.

## Materials and methods

### Materials and reagents

Calcium chloride (CaCl_2_, Catalog #C110768, American Chemical Society (ACS), ≥ 96.0%), Sodium carbonate anhydrous (Na_2_CO_3_, Catalog #S111737, ACS, 99.5%), CaCO_3_ powder (Catalog # C111984, 99%) and HEPES (Catalog #H109408) were purchased from Aladdin (Shanghai, China). The fluo-4 AM calcium indicator (Catalog #HY-101,896) was purchased from MedChemExpress (Shanghai, China). Lyso-Tracker Red (Catalog #C1046), Calcium Ion Assay Kit (Catalog # S1063S), and Hoechst 33,342 (Catalog #C1022) were purchased from Beyotime Biotechnology (Shanghai, China). ovalbumin (OVA, Catalog # A2512) was purchased from Sigma-Aldrich (Milwaukee, WI, USA). BODIPY™ 650/665-X NHS Ester (Catalog #D10001) was purchased from Thermo Fisher Scientific (Waltham, MA). Recombinant mouse Cytokines (GM-CSF and IL-4) were purchased from Peprotech. ELISA kits for TNF-α and IL-12p40 were purchased from Dakewei (Shenzhen, Guangdong, China). MACS® Tissue Storage Solution (130-100-008) was purchased from Miltenyi Biotec (Cologne, Germany). Anti-mouse PD1 (Catalog #CD279, clone RMP1-14) was purchased from Bio-X Cell Inc. The following antibodies were used: the purified anti-mouse CD16/CD32-Fc block, (clone 93, REF. NO.14-0161-86, eBioscience), PE-Cy7/APC-conjugated anti-mouse CD11c (clone N418, CAT. NO. 117,318/117,310, Biolegend), PE-conjugated anti-mouse CD86 (clone A17199A, REF. NO. 159,204, Biolegend), FITC-conjugated anti-mouse I-A/I-E (clone M5/114.15.2, REF. NO.11-5321-82, Invitrogen), Brilliant Violet 421™-conjugated anti-mouse I-A/I-E (clone M5/114.15.2, REF. NO. 107,631, Biolegend), PE-conjugated SIINFEKL/H-2Kb-reactive monoclonal antibody (clone eBio25-D1.16 (25-D1.16), REF. NO. 12-5743-81, eBioscience), APC-conjugated-anti-mouse CD3 (clone 17A2, REF. NO. 100,236, Biolegend), PE-Cy7-conjugated anti-mouse CD4 (clone RM4-5, REF. NO. 25-0041-82, eBioscience), FITC-conjugated anti-mouse CD4 (clone GK1.5, REF. NO. 100,406, Biolegend), PE-Cy7-conjugated anti-mouse CD8a (clone 53 − 6.7, REF. NO. 25-0081-81, eBioscience), FITC anti-mouse CD8a (clone 53 − 6.7, REF. NO. 100,706, Biolegend), PE-conjugated anti-mouse FOXP3 (clone MF-14, REF. NO. 126,404, Biolegend), PE-conjugated anti-mouse IFN-γ (clone XMG1.2, REF. NO. 505,808, Biolegend), Zombie Aqua™ Fixable Viability Kit (REF. NO. 423,101, Biolegend), Brilliant Violet 421™ anti-mouse CD69 (clone H1.2F3, REF. NO. 104,545, Biolegend).

### Cell culture

The DC2.4 cell line was provided by the Chinese Academy of Sciences cell library, cultured in RPMI-1640 supplemented with 10% FBS (Gibco), 2 mM L-glutamine, 100 U mL^− 1^ penicillin, 0.1 mg mL^− 1^ streptomycin (RPMI-1640 complete medium) at 37 ℃ with 5% carbon dioxide (CO_2_). Professor Ang Li kindly provided the B16-OVA cell line from the School of Life sciences and technology at Tongji University. The B16-OVA cells were cultured in RPMI-1640 complete medium and 400 µg mL^− 1^ G418.

### Synthesis of ANSs, LB, or BANSs

The preparation of antigen nanosponges (ANSs) with different sizes was performed based on our previous study [9]. CaCO_3_ powder (0.02 g) was first prepared in 5 ml plastic tubes. ANSs of approximately 40 nm at various pH were then poured along the wall of the plastic tube, followed by different stirring speeds at different temperatures over time. The product was washed several times with HEPES buffer (20 mM HEPES, 150 mM NaCl, pH 7.4). In this synthesis strategy, modulating pH in the reaction system was essential to obtain uniform BANSs. The ideal preparation of BANSs was carried out in ANSs with pH 6.5–7.0 at 120 rpm and 25 °C. The preparation of large size of BANSs (LB) was carried out in ANSs with pH 6.5–7.0 at 120 rpm and 25 °C.

### Synthesis of OVA@CaCO_3_

Preparation of OVA@CaCO_3_ was performed according to Shuang Wang et al [7]. Aqueous solutions of CaCl_2_ (0.5 M), Na_2_CO_3_ (0.5 M), and OVA (1 wt %) were first prepared as stock solutions. In a typical synthesis, a mixture of CaCl_2_ (500 µL) and OVA solution (20 mL) was prepared in a beaker and maintained under moderate stirring for 5 min. Subsequently, a solution of Na_2_CO_3_ (500 µL) was dropwise injected into the above-mixed solution under vigorous stirring. This system was stirred for 10 min, and the product was collected by centrifugation. All chemicals were of analytical grade and used without further purification.

To form FITC-labeled ANSs, LB, or BANSs, aqueous solutions of Sodium hydrogen carbonate (NaHCO_3_), FITC, and OVA were first prepared as stock solutions. FITC and OVA were mixed in carbonate buffer solution (pH 10) for 6 h at room temperature and then prepared as described above.

To form BIODIPY-labeled ANSs, LB, or BANSs, OVA was incubated with BODIPY™ 650/665-X NHS Ester for 1 h at room temperature and then prepared as described above.

### Characterization of OVA@CaCO_3_, ANSs, LB, or BANSs

The morphology of the samples was examined using transmission electron microscopy (TEM; JEOL JEM-1230 microscopes at 80 kV, JEM-1230, Japan). For TEM characterization, nanoparticle suspension was air-dried on gold grids. The ζ-potentials, intensity size distribution, and PDI of the nanoparticle were measured in Milli-Q water on a Zetasizer Nano-ZS90 instrument (Malvern Instrument, U.K.). UV-vis absorption measurements were carried out on a Cary 50 UVVIS-NIR spectrophotometer. Structural properties of the samples were also studied by X-Ray diffraction (XRD) using a Bruker XRD D8 Advance diffractometer in Bragg Brentano configuration for powders and a sweeping from 20 to 80. Thermogravimetric analysis (TGA) was performed on a Discovery TGA 55 instrument (TA Instruments, New Castle, USA). The functional groups of the samples were analyzed using a Fourier transform infrared spectrometer (FTIR) (Nicolet iS10, Thermo Fisher Scientific, USA) in the wavenumber range of 400–4000 cm^− 1^. The obtained samples were characterized using ICP-OES (Agilent 725, USA).

### Observation of CO_2_ generation in BANSs by ultrasound imaging

The generation of CO_2_ in BANSs was monitored using an ultrasound imaging system with an 8 MHz transducer (TELEMED, Lithuania). The BANSs were placed in a round-bottomed simulation tube mold. B-mode anatomic images automatically displayed the CO_2_ generation.

### Calcium ion assay

The dissolution of Ca^2+^ under different pH conditions was determined by an arsenazo III assay. First, 0.2 mM arsenazo III in HEPES buffer (20 mM HEPES, 150 mM NaCl, pH 7.4) was prepared. BANSs were immersed in buffer solutions (pH 5.0 or 7.4) (calcium-free solution) and shaken evenly at 37 °C; the Ca^2+^-released sample was obtained by centrifugation (12,000 rpm, 10 min). After mixing 50 µL samples with 1 mL of arsenazo III solution, the absorbance was measured at 656 nm. The calcium ion (Ca^2+^) concentration was measured using a Calcium Ion Assay Kit (Beyotime Biotechnology, Shanghai, China).

### In Vitro cytotoxicity studies

Cell viability of BANSs was assessed using DC2.4. After the cells were incubated with a series of nanovaccines ranging from 62.5 µg mL^-1^ to 300 µg mL^-1^ for 24 h, the cytotoxicity assessment was measured by CCK-8 analysis. Cell viability was calculated as follows: cell viability (%) = (sample OD - blank OD)/(negative control OD - blank OD) × 100%.

### Evaluation of antigen internalization

Cellular uptake of FITC-labeled OVA was measured using a flow cytometer (CytoFLEX LX, Beckman Coulter) after incubation with different formulations.

### Calcium production in vitro

DC2.4 was treated with BANSs (100 µg mL^-1^) at 37 °C for 6 h, then co-culturing with 10 µM Fluo-4 AM for 30 min. The production of calcium was immediately analyzed by flow cytometry.

### Lysosomal stability assay

DC2.4 cells were pre-treated with or without NH_4_Cl (an acidizing inhibitor, 20 mM) for 1 h, followed by incubation with OVA, ANSs, or BANSs (100 µg mL^-1^) for 6 h. The cells were collected and stained with Lyso-Tracker Red. The LysoSensor probe has a pKa of 5.2 and can identify endo-/lysosome. The fluorescent intensity of the Lyso-Tracker Red probe can be measured and evaluated by flow cytometry.

### Colocalization by confocal laser scanning microscopy

DC2.4 was incubated with OVA and BANSs for 6 h. Cells were gently washed with PBS to remove excess nanovaccines. Lyso-Tracker Red was added to the culture media to obtain a final concentration of 50 nM and incubated for 1 h following the supplier’s protocol for endo-/lysosome staining. Cells were gently washed with PBS and incubated with Hoechst 33,342 (1 µg mL^-1^) for 10 min to stain the nucleus. Finally, the cells were then analyzed under CLSM with a 60× oil immersion objective. Pearson’s correlation coefficient (PCC) values were obtained from ImageJ software.

### Animals

Female C57BL/6 mice (6 weeks old) were purchased from Shanghai Laboratory Animal Center (SLAC, Shanghai, China) and were housed in a sterilized, specific pathogen-free (SPF) Lab of Tongji University. All protocols performed on animals in this study were supported by the Animal Ethics Committee of Tongji University (Animal Ethics: TJAA07720104).

### Activation of bone marrow-derived dendritic cells in vitro

BMDCs were isolated from the femurs and tibias of 6-week-old female C57BL/6 mice and cultured in RPMI 1640 medium containing 10 ng ml^− 1^ IL-4 and 20 ng ml^− 1^ recombinant mouse granulocyte/macrophage colony-stimulating factor (GM-CSF) for 6 days [34]. The expression of costimulatory surface markers of BMDCs was measured by flow cytometry after staining with fluorescent antibodies. Otherwise, to monitor the cytokine profile of the BMDCs, cell culture supernatants were collected, and the secretion levels of IL-12p40 and TNF-α were detected using commercial ELISA kits according to the manufacturer’s instructions.

### In Vitro assays of OVA cross-presentation

BMDCs were generated from mouse bone marrow. On day 6, immature BMDCs were stimulated for 20 h with OVA, ANSs, BANSs, or SIINFEKL peptide with or without the pretreatment of NH_4_Cl and SIINFEKL peptide. Cells were stained using anti-mouse CD11c and monoclonal antibody 25d1.16 (Biolegend) and analyzed using flow cytometry.

### Nanoparticle distribution in lymph nodes

To observe the distribution of ANSs, LB, or BANSs in lymph nodes, BODIPY-labeled ANSs, LB, or BANSs were subcutaneously injected into the footpad of mice. The popliteal lymph nodes in different groups were imaged and quantified by an Aniview system (excitation filter: 630 nm, emission filter: 680 nm). BODIPY-labeled ANSs, LB, or BANSs were subcutaneously injected into the hock of mice. Mice were sacrificed at 24 h, and inguinal lymph nodes were collected for a frozen section study. The lymph nodes were washed with PBS, embedded with tissue freezing medium, and sectioned using a freezing microtome (Leica CM1950, Germany). They were fixed with 4% paraformaldehyde for 10 min, washed with PBS, and stained with 4’,6-diamidino-2-phenylindole (DAPI), APC-CD11c, and FITC-CD11b for 15 min. They were observed using a confocal laser scanning microscope.

To verify that Distinct APCs resident internalized ANSs, LB, or BANSs in lymph nodes, BODIPY-labeled ANSs, LB, or BANSs were injected into the hock of mice which were sacrificed at 24 h (*n* = 3 animals per group). Their inguinal draining lymph nodes were harvested, and single-cell suspensions were prepared and then passed through a 70 μm cell sieve. Cells were washed with PBS, surface‐stained with anti‐mouse CD11c and CD11b, and analyzed using a CytoFLEX flow cytometer (Beckman Coulter).

To investigate the ability of ANSs, LB, or BANSs to activate distinct APCs maturation, cross-presentation, and subsequent immune responses in vivo, different groups were subcutaneously injected into the hock of the mice (*n* = 3 animals per group). Three days after administration, dLNs were isolated and homogenized into a single-cell suspension. Then, the obtained cells were stained with anti-mouse CD11c, CD11b, CDF4/80, MHC-II, CD86, and monoclonal antibody 25d1.16 (Biolegend) and analyzed using a CytoFLEX flow cytometer (Beckman Coulter). At day 6 postvaccination, the lymph nodes were washed with PBS, embedded with tissue freezing medium, and sectioned using a freezing microtome (Leica CM1950, Germany). They were fixed with 4% paraformaldehyde for 10 min, washed with PBS, and stained with CD4 and CD8 for 1 h at 37 °C. They were observed using a confocal laser scanning microscope.

### Antitumor studies

B16-OVA tumor cells (2.0 × 10^5^ cells mouse^− 1^) were subcutaneously implanted into the flank of mice on day 0. Then, mice were treated as described in the relevant figure legend. Tumor volume (V) was calculated using V = WL^2^/2 (where W means the longest diameter and L represents the shortest diameter). On day 7, when palpable tumors appeared, mice were randomly divided into 5 groups (5 mice per group) and were respectively injected with ANSs, LB, BANSs^low^ (150 µg OVA + 45.06 µg CaCO_3_), or BANSs^high^ (150 µg OVA + 92.44 µg CaCO_3_). C57BL/6 mice were vaccinated (on day 7) via subcutaneous injection with various formulations (three times at 6-day intervals). On day 21 post-B16-OVA cell injection, tumor tissues were collected and triturated into cell suspension, stained with (1) APC-CD3, FITC-CD8, and PE-IFN-γ to label CTLs; (2) APC-CD3, PE-cy7-CD4, and PE-Foxp3 to label Tregs. Lymph nodes were collected and triturated into cell suspension, stained with (1) APC-CD3, FITC-CD8, and PE-IFN-γ to label CTLs; (2) APC-CD3, FITC-CD4, and PE-IFN-γ to label Th_1_ cells. For the mice sacrificed during the treatment, the dissected tissues were immediately harvested from mice directly into cold tissue storage solution (MACS) until processing. The main organs were collected for H&E staining.

### Combination cancer immunotherapy

First, live B16-OVA cells (2.0 × 10^5^ cells mouse^− 1^) were injected subcutaneously into the right flank of female C57BL/6 mice. The C57BL/6 mice (6–8 weeks old) were randomly divided into four groups (*n* = 6) and immunized as follows: four groups of the mice were subcutaneously injected with PBS, αPD-1 (2.5 mg kg^− 1^), BANSs (150 µg OVA + 92.44 µg CaCO_3_) or BANSs (150 µg OVA + 92.44 µg CaCO_3_) + αPD-1 (2.5 mg kg^− 1^). For the analysis of memory T cells, circulating peripheral blood mononuclear cells from the peripheral blood, lymph node, and spleen were harvested from the survived mice and stained with anti-CD3-APC, anti-CD8-FITC, anti-CD4-FITC, anti-CD44-PE, or anti-CD69-Brilliant Violet 421™. Data analysis was carried out using FlowJo software. Inguinal lymph nodes were collected for a frozen section study. The tumor and spleen were washed with PBS, embedded with tissue freezing medium, and sectioned using a freezing microtome (Leica CM1950, Germany). They were fixed with 4% paraformaldehyde for 10 min, washed with PBS, and stained with 4’,6-diamidino-2-phenylindole (DAPI), CD3, CD56, B220, CD8, PD-1, and PD-L1for 1 h at 37 °C. They were observed using a confocal laser scanning microscope.

### Statistical analyses

The data were analyzed using GraphPad Prism 8 software (GraphPad Software). Shapiro-Wilk normality test or Kolmogorov-Smirnov test were used to detect whether the data obeyed a normal distribution. The analysis showed all the experimental data obey the normal distribution and were suitable for the Student’s t-test, one-way analysis of variance (ANOVA), or Brown-Forsythe and Welch ANOVA test. One-way ANOVA or Brown-Forsythe and Welch ANOVA test were used for multiple comparisons when more than two groups were compared, and Student’s t-test was used for two-group comparisons. The threshold for statistical significance was P < 0.05, where one-way ANOVA was used. A P value < 0.05 was considered statistically significant and significance was labeled as *P < 0.05, **P < 0.01, ***P < 0.001, and ****P < 0.0001.

## Electronic supplementary material

Below is the link to the electronic supplementary material.

Supplementary Material 1

## Data Availability

The datasets used and/or analyzed during the current study are available from the corresponding author upon reasonable request.
