# Supplementary material for: CaCO3 powder-mediated biomineralization of antigen nanosponges synergize with PD-1 blockade to potentiate anti-tumor immunity
Source: J Nanobiotechnology. 2023 Apr 7;21:120. doi: 10.1186/s12951-023-01870-x (PMC10080855; doi:10.1186/s12951-023-01870-x)
Supplement: Supplementary file 1 — Supplementary Material 1 [file 12951_2023_1870_MOESM1_ESM.docx]

Supporting Information

CaCO_3_ Powder-Mediated Biomineralization of Antigen Nanosponges Synergize with PD-1 Blockade to Potentiate Anti-tumor Immunity

Runping Su, Jingjing Gu, Juanjuan Sun, Jie Zang, Yuge Zhao, Tingting Zhang, Yingna Chen, Gaowei Chong, Weimin Yin, Xiao Zheng, Bingbing Liu, Li Huang, Shuangrong Ruan, Haiqing Dong, Yan Li, and Yongyong Li*

Supporting Figures


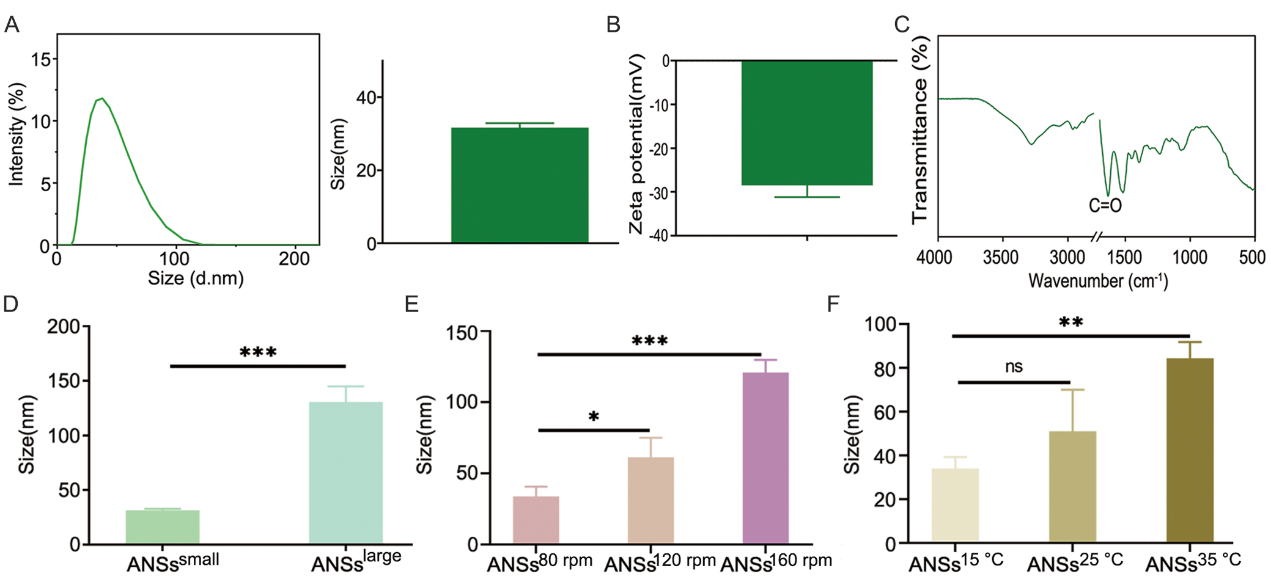


**Figure S1.** (A) The hydrodynamic size of ANSs. (B) The surface zeta potential of ANSs.

(C) Fourier-transform infrared spectroscopy (FT-IR) spectra of ANSs. (D) Different sizes of ANSs. (E) Particle size of ANSs at different shaker speeds. (F) Particle size of ANSs at different shaker temperatures. Error bars, mean ± SD. (*n* = 3). (ns, not significant, **P* < 0.05, ***P* < 0.01, and ****P* < 0.001).


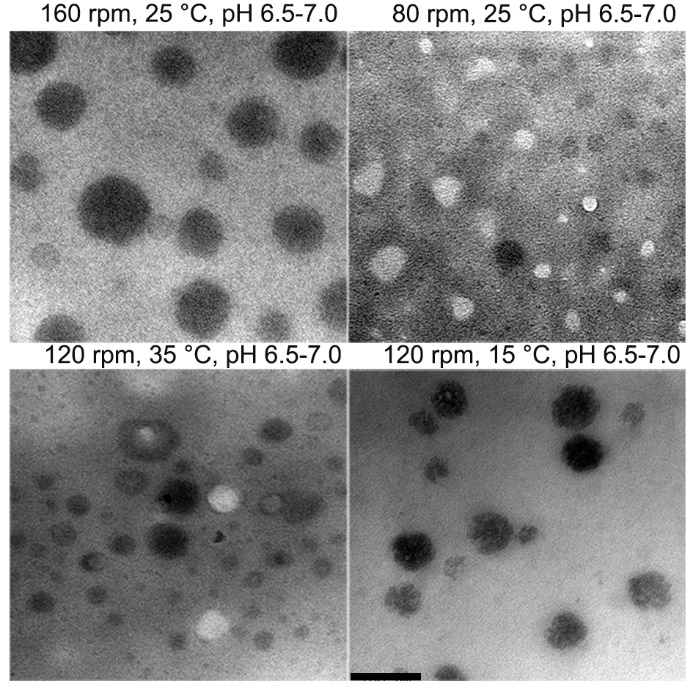


**Figure S2.** Representative TEM images of BANSs at different preparation conditions. The scale bars represent 0.2 µm.


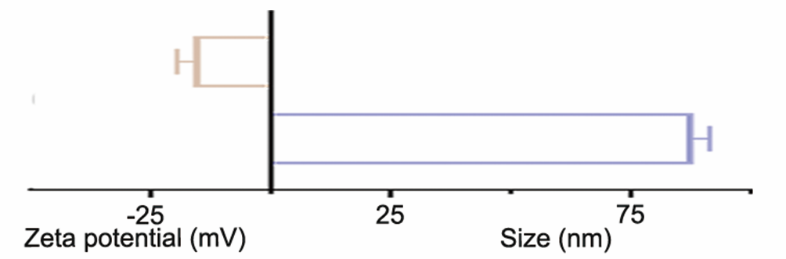


Figure S3. The hydrodynamic size and the surface zeta potential of BANSs.


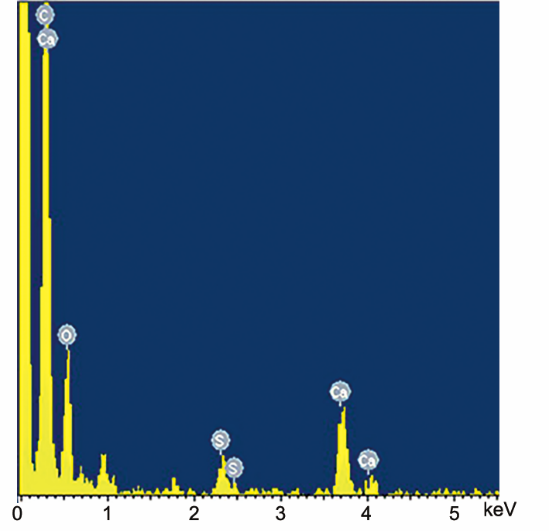


**Figure S4.** Energy-dispersive X-ray spectroscopy (EDX) elemental mapping of BANSs.


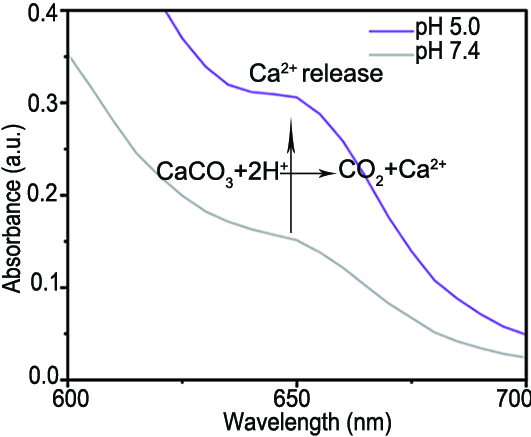


**Figure S5.** The UV absorption spectrum of calcium-arsenazo III. Ca^2+^ release from the BANSs dispersed in buffer solutions at different pH values.


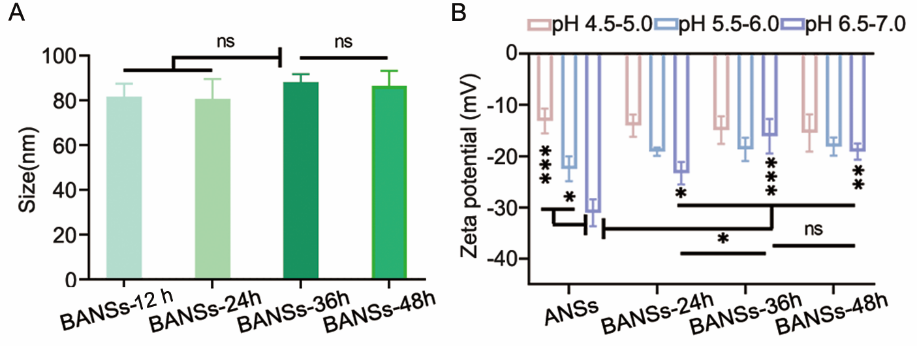


**Figure S6.** (A) The hydrodynamic size of BANSs for various times and (B) The surface zeta potential of ANSs and BANSs for various times. Error bars, mean ± SD (*n* = 3). (ns, not significant, **P* < 0.05, ***P* < 0.01, and ****P* < 0.001).


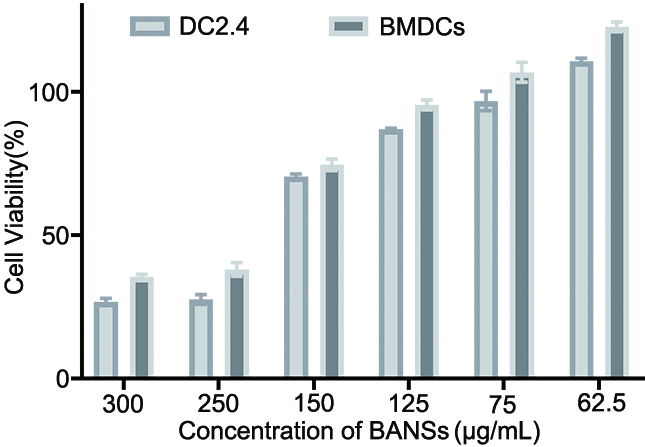
`

**Figure S7.** Cell-viability assay in DC2.4 and BMDCs incubated with BANSs. Error bars, mean ± SD. (*n* = 3).


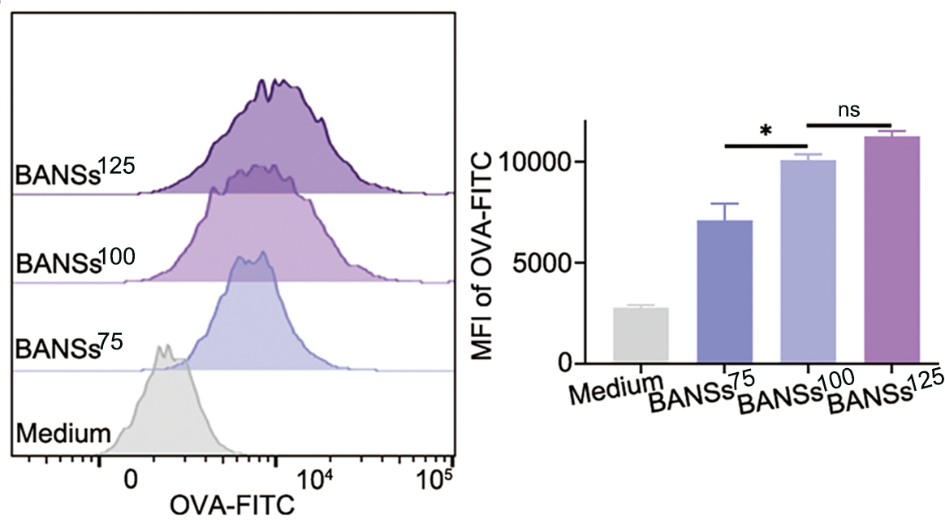


**Figure S8.** (A) Representative flow cytometry histograms and (B) the mean value analysis of dose-dependent internalization of BANSs after incubation with different concentrations. Blank, DC2.4 cells without any treatment. Error bars, mean ± SD (*n* = 3). (ns, not significant, **P* < 0.05).

**
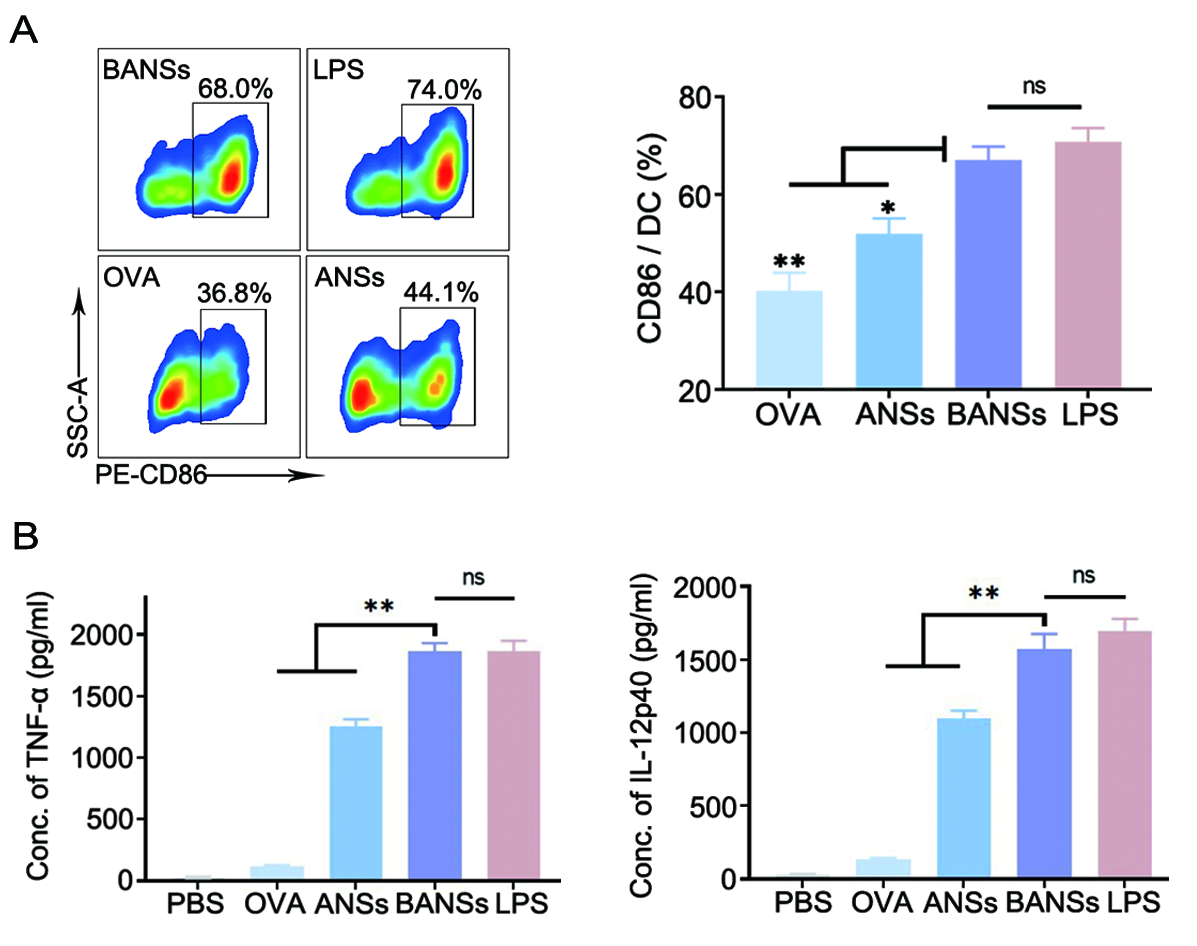
**

**Figure S9.** TNF-α and IL-12p40 secretion by BMDCs. Error bars, mean ± SD (*n* = 3). (ns, not significant, **P* < 0.05, ***P* < 0.01, *****P* < 0.0001).

**
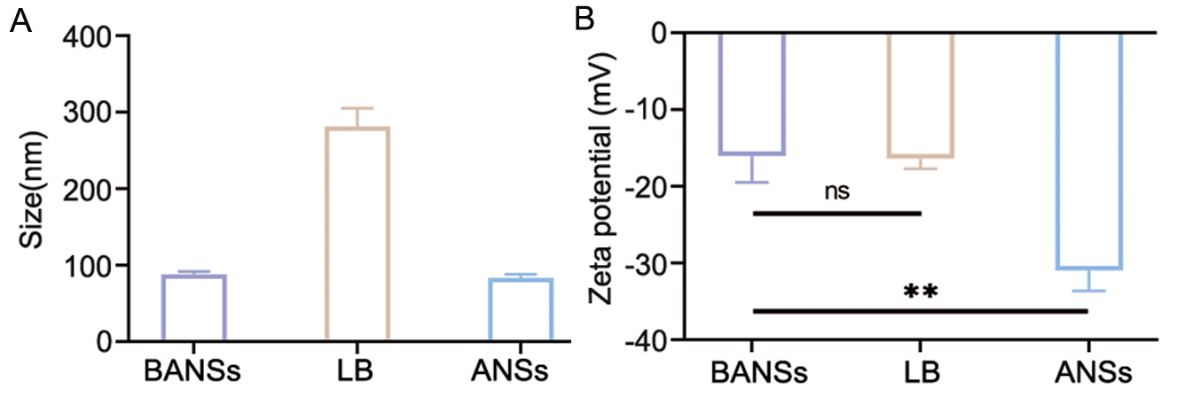
**

**Figure S10.** Size and Zeta-potential of ANSs, LB, and BANSs, Error bars, mean ± SD. (*n* = 3). (ns, not significant, ***P* < 0.01).


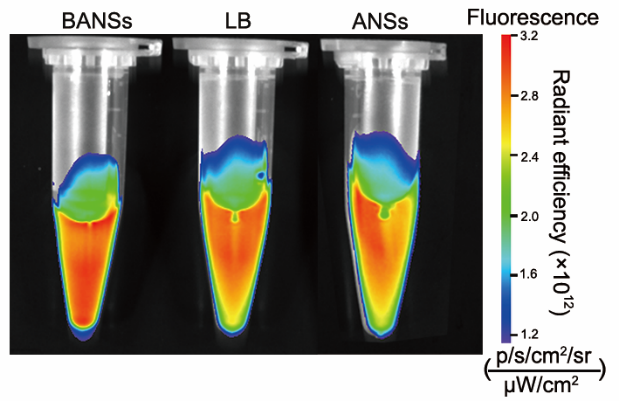


**Figure S11.** Fluorescent images of nanovaccines labeled with BODIPY.


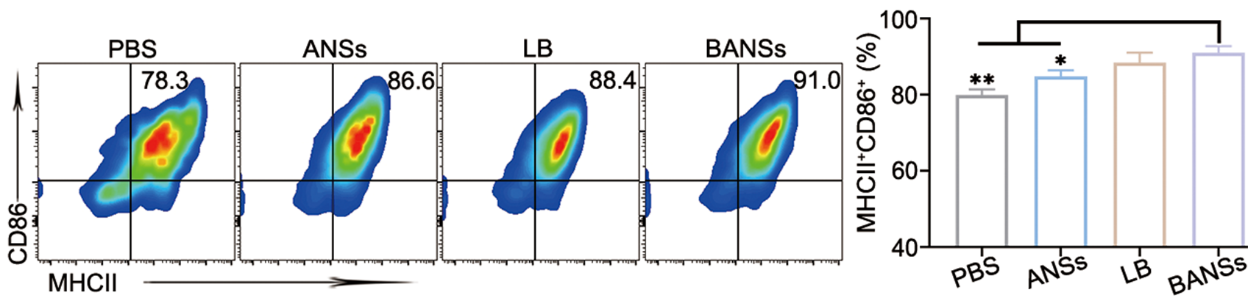


**Figure S12.** Analysis of maturation of macrophages treated with BANSs. Error bars, mean ± SD. (*n* = 3). (**P* < 0.05, ***P* < 0.01).


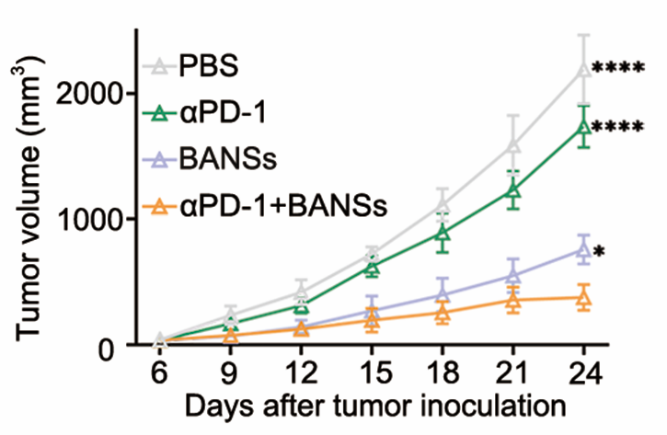


**Figure S13.** Tumor growth curves.

**
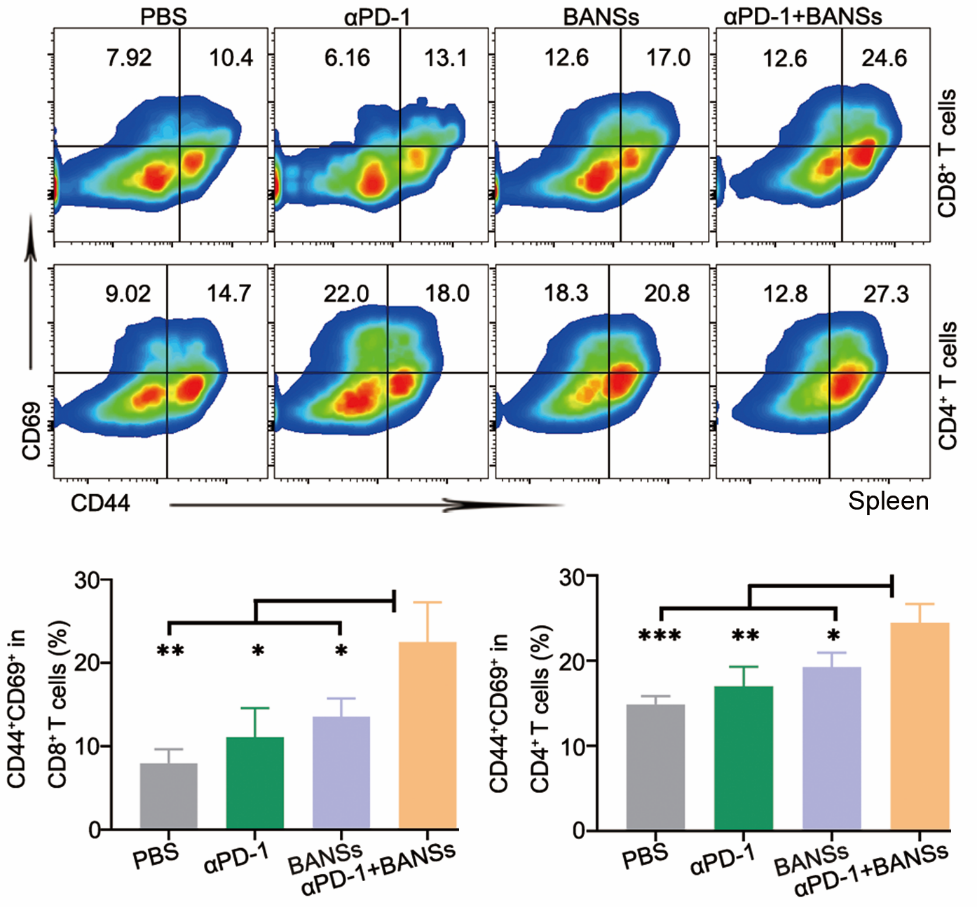
**

**Figure S14.** (A) Representative FACS plots of CD44 and CD69 on T cells following gating for CD8^+^ and CD4^+^ cells in spleen tissues (*n* = 4 to 6 mice per group). (B) Number of CD44 and CD69 on T cells following gating for CD8^+^ cells in spleen tissues. (C) Number of CD44 and CD69 on T cells following gating for CD4^+^ cells in spleen tissues (*n*= 4 to 6 mice per group). Error bars, mean ± SD. (**P* < 0.05, ***P* < 0.01, and ****P* < 0.001).


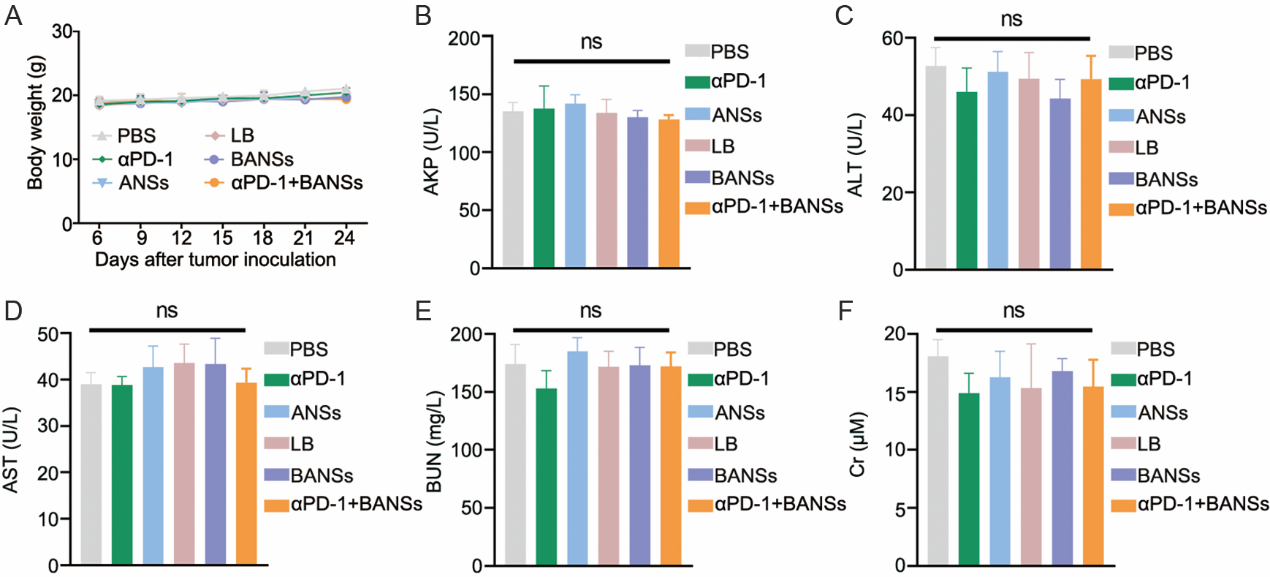


**Figure S15.** (A) Average body weight of mice in different treatment groups, measured every 2 days. Data are shown as means ± SD from five independent animals. The systemic toxicity of mice after different treatments analyses of serum function markers of the liver (alkaline phosphatase [AKP] (B), alanine aminotransferase [ALT] (C), and aspartate aminotransferase [AST] (D) and kidney (blood urea nitrogen [BUN] (E) and creatinine [Cr] (F). The data are presented as means ± SD (*n* = 3). (ns, not significant).


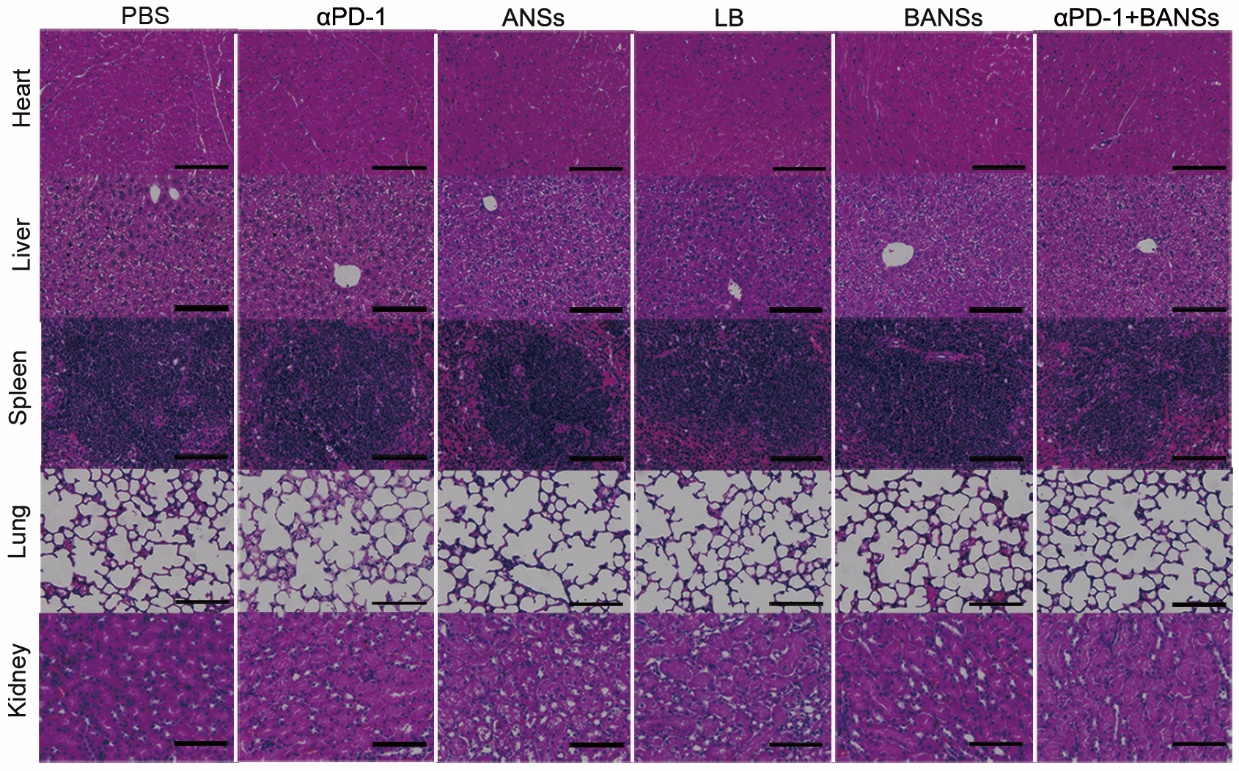


**Figure S16.** H&E staining of main organ sections at the end of treatment. The scale bars represent 100 µm.
